# Diversification, Evolution and Sub-Functionalization of 70kDa Heat-Shock Proteins in Two Sister Species of Antarctic Krill: Differences in Thermal Habitats, Responses and Implications under Climate Change

**DOI:** 10.1371/journal.pone.0121642

**Published:** 2015-04-02

**Authors:** Kévin Cascella, Didier Jollivet, Claire Papot, Nelly Léger, Erwan Corre, Juliette Ravaux, Melody S. Clark, Jean-Yves Toullec

**Affiliations:** 1 Sorbonne Universités, UPMC Université Paris 06, UMR 7144 CNRS, Equipe ABICE, Station Biologique de Roscoff, 29680 Roscoff, France; 2 CNRS, UMR 7144, Adaptation et Diversité en Milieu Marin, Station Biologique de Roscoff, 29680 Roscoff, France; 3 Université de Lille1, CNRS UMR8198, Ecoimmunology of Marine Annelids, 59655 Villeneuve d’Ascq, France; 4 Sorbonne Universités, UPMC Université Paris 06, UMR 7208 CNRS, Equipe AMEX, 75005 Paris, France; 5 CNRS 7208, BOREA, UPMC Université Paris 06, 75005 Paris, France; 6 Sorbonne Universités, UPMC Université Paris 06, FR 2424 CNRS, ABiMS, Analysis and Bioinformatics for Marine Science, Station Biologique de Roscoff, 29680 Roscoff, France; 7 CNRS, FR 2424, Station Biologique de Roscoff, 29680 Roscoff, France; 8 British Antarctic Survey, Natural Environment Research Council, High Cross, Madingley Road, Cambridge, CB3 0ET, United Kingdom; University of Ferrara, ITALY

## Abstract

**Background:**

A comparative thermal tolerance study was undertaken on two sister species of Euphausiids (Antarctic krills) *Euphausia superba* and *Euphausia crystallorophias*. Both are essential components of the Southern Ocean ecosystem, but occupy distinct environmental geographical locations with slightly different temperature regimes. They therefore provide a useful model system for the investigation of adaptations to thermal tolerance.

**Methodology/Principal Finding:**

Initial CT_max_ studies showed that *E*. *superba* was slightly more thermotolerant than *E*. *crystallorophias*. Five Hsp70 mRNAs were characterized from the RNAseq data of both species and subsequent expression kinetics studies revealed notable differences in induction of each of the 5 orthologues between the two species, with *E*. *crystallorophias* reacting more rapidly than *E*. *superba*. Furthermore, analyses conducted to estimate the evolutionary rates and selection strengths acting on each gene tended to support the hypothesis that diversifying selection has contributed to the diversification of this gene family, and led to the selective relaxation on the inducible C form with its possible loss of function in the two krill species.

**Conclusions:**

The sensitivity of the epipelagic species *E*. *crystallorophias* to temperature variations and/or its adaptation to cold is enhanced when compared with its sister species, *E*. *superba*. These results indicate that ice krill could be the first of the two species to be impacted by the warming of coastal waters of the Austral ocean in the coming years due to climate change.

## Introduction

Although global warming is affecting the whole planet, the effects are highly variable and regional. Certain areas, notably, the high latitude regions, are currently experiencing rapid rates of climate change, as shown by reductions in the duration of the Arctic sea ice and also the temperature increases along the Antarctic Peninsula, where air temperatures have risen by +3°C and the surface waters by +1°C in the past 50 years [1,2]. Although these increases in temperature seem modest when compared to those experienced in temperate regions, they represent a real threat for the endemic organisms, particularly those in the Antarctic, which have evolved in a very cold and stable environment for 15 to 25 million years [3]. This has resulted in a marine fauna with some unique adaptations to the cold, including a loss in their capacity to manage the variations of temperature in their environment via the cellular stress response (CSR)[4].

The environmental impacts of climate change and its effects on species are complex. Thus predicting the outcomes of the application of even a single stressor is challenging. One approach is to study of the direct effect of temperature on the organism itself. This enables estimating, not only of the capacities of the response to thermal shock but also to understand the associated physiological mechanisms. The latter should include the study of phenotypic plasticity and acclimation, which may modify thermal tolerance capacities [5], but also the adaptive potential of the species, inherent in the genetic diversity at the population level, which provides the material for the emergence of better adapted mutations [6]. Another different, but complementary approach is based on the fact that the geographic distribution of ectothermic eukaryotes is partly determined by temperature. This can be seen in the distribution of boreal marine species across a latitudinal thermal gradient and their vertical migration along this gradient [7–9]. Therefore evaluating the evolutionary history of each species and understanding the physiological mechanisms which underlie this temperature dependant separation can help explain not only the actual distribution of these species, but also predict how global warming can impact these species in terms of their location and their capacities to deal with environmental change [10,11]. For this type of study it is important to choose ecotypes of cosmopolitan species and/or a complex of phylogenetically closely related species, which present clearly demarked distributions, based on different thermal properties, as study species.

Using this rationale, a comparative thermal tolerance study was undertaken on two sister species of Euphausiids (Antarctic krill), *Euphausia superba* and *Euphausia crystallorophias* [12,13]. *Euphausia superba* or Antarctic krill *sensu stricto*, lives in huge swarms beyond the continental shelf and is well known as an important food source for the region’s charismatic higher predators [14]. In addition it is an increasingly profitable fisheries resource [15,16]. *Euphausia crystallorophias* also lives largely in swarms but its distribution is restricted to the continental plateau with a life cycle reliant on the presence of sea ice, hence the common name of ice krill [17]. *E*. *crystallorophias* has been less well studied, but its trophic importance is equally fundamental to the continental plateau zone, where it replaces its sister species at depths above 500 m. So, although both species are critical for the equilibrium of the Southern Ocean ecosystem because they represent the most abundant zooplankton biomass in the food chain in this region [18–20], their geographical distributions do not overlap [21]. This geographic separation may be related to the abiotic profiles of each particular habitat and potentially impacts their physiological functioning. Temperature is a strong candidate for one of the segregating factors influencing the separation of the two species, as one is epipelagic, coastal (*E*. *crystallorophias*), the other is deep sea pelagic (*E*. *superba*). The intrinsic relationship of ice krill with the low temperatures of the continental plateau over millions of years and also with sea ice, means that this potentially strictly stenothermal species could be particularly vulnerable to warming of the Southern Ocean. In contrast, the density of Antarctic krill north of the Peninsula and around South Georgia seems to vouch for a temperature tolerance of the order of several degrees above zero [15,22]. Hence, they represent a pertinent model system for the investigation of adaptations, specifically related to thermal tolerance.

This study had three objectives: Firstly to determine the upper lethal temperature limits *in vivo* (CT_max_) of each krill species (at 0.1°C per minute). Secondly to estimate the lower limit of the first onset of a thermal stress response by measuring the expression of their heat shock response. Most organisms have the potential to manage the thermal fluctuations in their environment via highly conserved cellular defence mechanisms, the best known of which consists of the induction of a complex of heat shock proteins (Hsp) [23]. In stressful situations, the Hsps are known to play an essential role in the prevention of protein aggregates and in the conservation of the quaternary structure of proteins. Within the different families of Hsps, the 70 kDa forms (Hsp70s) are the most highly conserved family and are the best studied. *Hsp70* gene family members were identified and characterised in each sister species. The final aim was to conduct a phylogenetic analysis of the different characterised isoforms of Hsp70 in order to establish the relationships of paralogy and orthology between the two species, also to conduct analyses to estimate their evolutionary rates and to test the hypotheses that diversifying selection has contributed to the diversification of this gene family and to the ecological specialisation of the two species.

## Material and Methods

This project (IPEV- 1039) was approved by IPEV (Institut Paul Emile Victor, the French Polar Institute) review committee and was declared to and approved by the Terres Australes et Antarctiques Françaises in 2009 according the Annex I of the Madrid Protocol and the French Decret No 2005–403. No endangered or protected species were used.

### Animal collection

The two species of krill were fished off the French base Dumont d’Urville (DDU) in Terre Adélie either from the continental plateau (*Euphausia crystallorophias*) or just beyond the same plateau (*E*. *superba*). Fishing was carried out using an Isaacs-Kidd midwater trawl (IKMT) from the ship L’Astrolabe at a maximum speed of 2 knots for 10–15 minutes. The sampling depth was determined by sonar observations of swarms, taking samples from those located between 20 and 50 metres. After fishing the animals were moved to a flow-through tank on board, which was continuously fed with water at -0.5–+0.5°C. Both species were acclimated to conditions in different main aquariums, where a circulation pump was used to set up a vertical circulation current to maintain the animals in the water column prior to experimentation and minimise holding stress.

### Critical temperature estimation

The protocol was strictly identical for the two species with n = 130 for *E*. *crystallorophias* and n = 43 for *E*. *superba*. Sampling sizes depended on the quantities of harvested animals the day before the experiment. After around 24 hours in the main aquarium, the swimming animals were selected and put into two aquariums containing seawater at the same temperature as the main stock aquarium. These aquariums were placed within a larger tank containing fresh water. A copper coil linked to a thermostat and pump was run through the system. The coil was used to pump hot water around the system to warm up the tank water for the critical temperature experiments. A circulating pump ensured that the warming was equally distributed around the tanks and air pumps were used to maintain the oxygen levels in the aquariums and also facilitate water mixing. The rate of temperature increase in this experiment was 1°C every 10 minutes. The rate of temperature change was chosen according to the pre-existing literature and the technical limits of our aquarium system. The animals were maintained in the tank until they were no longer able to escape tactile stimuli of the probing rod. At this point, it was considered that the temperature limit had been reached and the animals were taken out of the aquarium and snap frozen in liquid nitrogen. The CT_max_ was considered as the temperature at which survival of the experimental animals reached 50%. It was determined though the non-linear curve fitting option in JMP10 (SAS). The survival curve we used was: Survival = c/(1+(T/CT_max_) ^b^) where c is the plateau value before the sharp decrease, CT_max_ the temperature at which 50% of mobile animals is reached, and b a sigmoidicity coefficient. The program explores the different values of these three parameters, and calculates a Chi-square. While exploring the different parameter values, the program aims at minimizing this Chi-square and converges towards a value for each parameter (provided with a standard error).

### Thermal shocks

The same aquarium system was used for the longer thermal shocks, 3 or 6 hours at +3°C or +6°C. The animals were put directly into the warmed water. At the same time a set of control animals were removed from the main aquarium and kept in aquarium at 0°C for 3 or 6 hours as well. These time exposures have been chosen to highlight the potential regulation on the *Hsp70* mRNA expression during and after the shock duration, even in the case of a late response. After removal from the tanks, experimented and control animals, about 10 per condition, were snap frozen in liquid nitrogen.

### Characterisation of Hsp70 isoforms and cDNA cloning

The different HSP70 isoforms were initially characterised in the two species from transcriptome data, generated using 454 technology for *E*. *superba* [24](SRA023520) and Illumina sequencing for *E*. *crystallorophias* [25](EMBL-EBI: ERP002510). Sequences were confirmed using isolation of the relevant cDNAs and sequencing. All samples were transported to the Roscoff Biological Station at -80°C. Total RNA was extracted from abdomen of each frozen individual using the RNeasy kit (Qiagen), according to manufacturers instructions. Concentrations of total RNA were determined using a Nanodrop by measuring ultraviolet absorbance at 260nm. RNA purity was checked by determining the A260/A280 ratio and checking the integrity on an agarose gel. Then 1μg of mRNA was retrotranscripted using the M-MLV Reverse transcriptase kit (Affimetrix/USB) according to manufacturer’s instructions using SKdT primers to generate total single stranded cDNA from each sample.

The Hsp70 isoforms were PCR amplified from the cDNA using specific primers. Four pairs of PCR primers were used for each gene in order to clone each isoform in overlapping 1000 bp sections to facilitate full depth sequencing of the whole gene. PCR products were gel purified and Hsp70 fragments were inserted into the pGEM-T vector (Promega Corporation, USA). Plasmids were transformed into DH5α bacteria (*Escherichia coli*; Life Technologies^TM^, USA). Transformed bacteria were selected and PCR verification carried out on positive clones. Plasmids were then extracted and sequenced with same primers as before. These sequences were used to confirm the Illumina gene assemblies and the Hsp70 contigs.

### qPCR analysis

mRNA levels of the Hsp70 isoforms were determined by quantitative RT-PCR amplification, on 6 to 10 individuals for each heat shocks and time condition. Q-PCR reactions were performed in a 5μL volume containing 2.1μl of each diluted reverse transcription product (1:200), 0.4 μM of each specific primer and 2.5μL of SYBR Green I master mix (Roche, France). The amplification was carried out as follows: 95°C for 15 min, then 55 cycles of 95°C for 10 sec and 60°C for 30 sec. A dissociation curve was generated and PCR efficiency was estimated for each primer pair. All primer pairs tested generated a single peak in the dissociation curve and a PCR efficiency of 80–100%. Data were analysed with the LightCycler 480 software. The 18S gene was chosen as a reference gene using the BestKeeper algorithm [26] after testing EF1α, 18S, RPL8, and GAPDH as potential normalising housekeeping sequences. Hsp70 expression was subsequently normalized to this reference. A Kruskall–Wallis test and a Dunn’s multiple comparison test of the mean normalized expression of the Hsp70 isoform genes were performed to determine significant differences between control and heat shocked samples.

### Phylogenetic reconstruction and molecular clock tests

Phylogenetic reconstructions were carried out on 54 HSP70 proteins from different species including the krill isoforms, using Bayesian Inference (BI) and Maximum Likelihood (ML) methods. Bayesian analysis was performed using MrBayes 3.1.2 with four chains of 10^6^ generations, trees sampled every 100 generations, and the burnin value set to 20% of the sampled trees. Protein sequences were analysed with a mixed amino-acid model [27]. Maximum likelihood reconstructions were carried out on amino-acid sequences, using PhyML [28,29] with an evolutionary model selected via Akaike Information Criterion with ProtTest (LG+G+F)[30], and validated with 100 bootstrap replicates. Insect Hsp70 (*Locusta migratoria*, *Manduca sexta*, *Bombyx mori*) sequences were used as out-groups.

The position of the paralog E within the phylogeny was inconclusive and depended upon the type of phylogenetic reconstruction that was carried out (ie. Bayesian vs ML reconstructions). The CodeML program within PAML vs 3.44 [31] was used in tandem with the model of codon substitutions (Goldman and Yang, 1994) to test between different tree topologies with the paralog E using a restricted alignment of the Hsp70 paralogs in the two species of krill (*Euphausia superba* and *E*. *crystallorophias*) only. The likelihood values obtained were then compared between the different trees using a KH normal test [32] and the Shimodaira and Hasegawa correction [33]. Both global and local clock calibrations (three molecular rates for the cytosolic (ABCE), mitochondrial (D) and GRP78 clades) were fitted to the tree according to Yang and Yoder [34] to test whether ancestral sequence reconstruction lead to different distributions in mutations between branches leading to the two krill species.

### Investigation of the branches/codons under positive selection

The search for branches and codons under positive selection between paralogs of Hsp70 and, more specifically between terminal branches leading to the two krill species within each paralog was performed using the software PaML v3.44 using CodeML and LRTs (Likelihood Ratio Test) between codon models. We tested whether Hsp70s evolved under different selective constraints across lineages by comparing the one ω (dN/dS) ratio neutral model (M0) and the free ω ratio branch model (one ω for each branch). We then identified codon sites that had experienced a positive change by comparing the M_3_ model (3 classes of ω_:_ ω _0_<1, ω _1_ = 1 and ω _2_>1) with the nearly neutral model M_1A_ (2 classes of ω: ω _0_<1 and ω _1_ = 1). Terminal branches for which selection was relaxed or positive were also tested for the presence of codons under positive selection in, at least, one of the two krill lineages using a branch-site model M_2A_ of selection and the investigated branch as the foreground lineage. This model includes four categories of ω (ω <1, = 1, ω >1 using either an average ω <1 or ω = 1 as a background). The significance of this model was evaluated using LRT against a null model, the ‘nearly neutral’ site model M_1A_. The Bayes empirical Bayes (BEB) algorithm was also used to compute posterior probabilities for the 3 classes of ω and to identify sites under selection in case of significant LRT.

### Investigation of the ancestral characters in the HSP70 sequences

An empirical Bayesian reconstruction of ancestral sequences at each internal node of the paralogous tree was carried out following the likelihood-based approach developed by Koshi & Goldstein [35].

## Results & Discussion

### Estimation of upper lethal temperature limits

**Fig 1:**
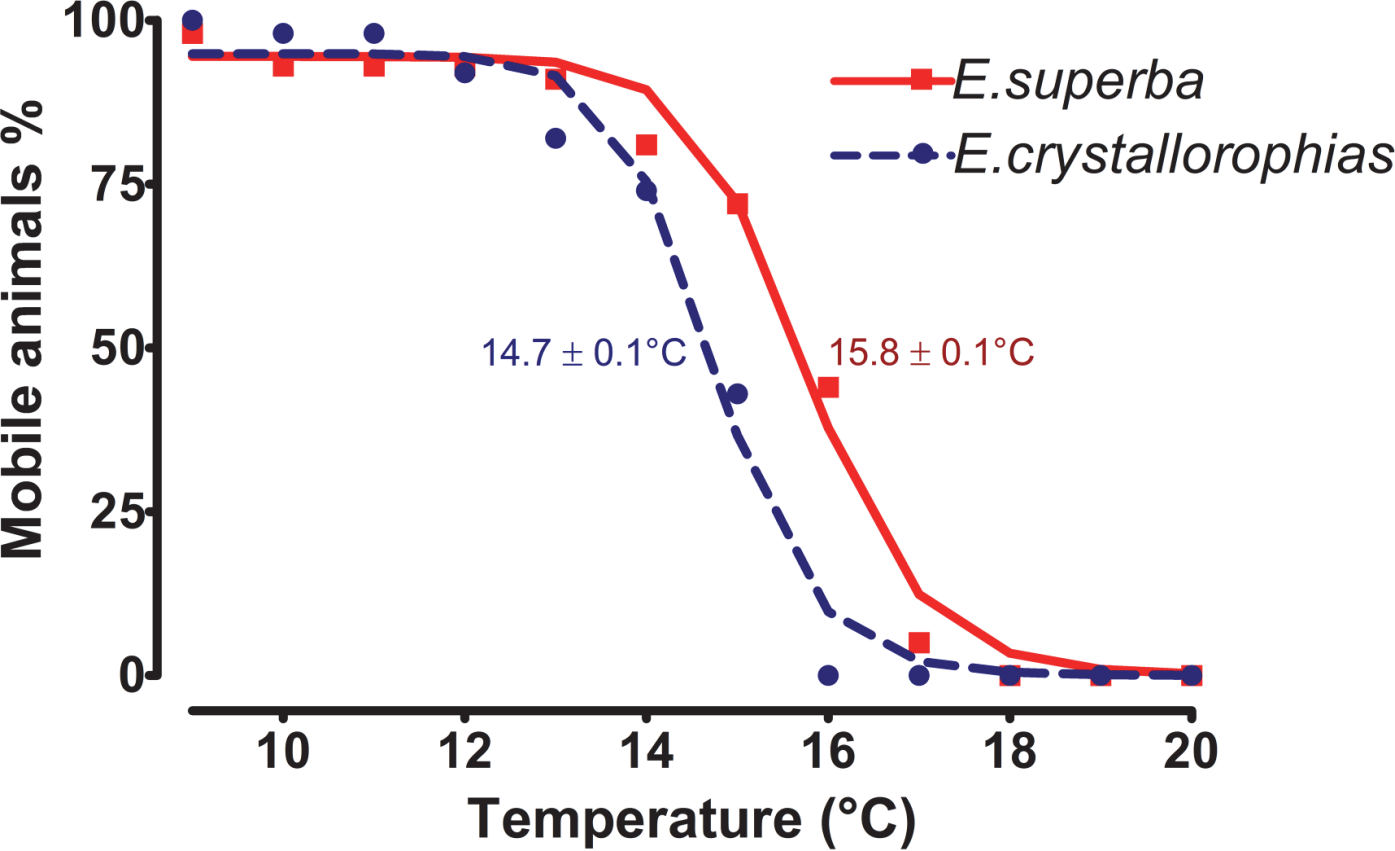
Curves representing the loss of mobility of a population of krill subjected to gradual temperature increase (0.1.min^-1^) (*E*. *crystallorophias* n = 130 blue curve; *E*. *superba* n = 43 red curve). CT_max_ is the temperature at which 50% of mobile animals are reached.

As a preliminary analysis to understand the thermal resilience of *E*. *superba* and *E*. *crystallorophias* in the Southern Ocean, short term acute exposures were undertaken, using a temperature increase of 0.1°C/min. The interpretation of the CT_max_ curves of the two species (Fig. 1) gives values for *E*. *superba* and *E*. *crystallorophias* of 15.8 ±0.1°C and 14.7 ± 0.1°C respectively. These temperatures indicate that these two species of krill may be some of the most thermotolerant invertebrates in the Southern Ocean [36–38]. These animals are particularly mobile, (pelagic and epi-pelagic) and whilst most measures to date have been carried out on benthic animals, studies have shown mobility to significantly correlate with thermal tolerance under rapid thermal challenges [38]. Whilst the thermal shocks applied here are relatively short and represent very rapid temperature increases, longer term experiments, especially for pelagic species, such as krill are difficult to put in place without a specialised facility and detailed knowledge of long-term husbandry, however, such short term studies can be used to extrapolate predictions for longer term survival [38]. There was an additional observation with this experiment, which may indicate that behaviour may also impact on the thermal response of these species. All animals for the experiments were taken from the upper water column of the stock aquarium ensuring that they were active swimmers, but there was an interesting difference in response between the two species. Despite the small difference in CT_max_ (1°C) between these two species, *E*. *superba* appeared to maintain a superior swimming capacity during the experiment. When the water temperature was only raised to 2°C, the ice krill response was characterised by swimming along the bottom (S1 Fig.). This may have been due either to a reduction in capacity indicated by a loss of swimming activity or simply a escape response to keep as deep as possible in the face of this environmental challenge [39,40].

### Identification and characterisation of Hsp70 sequences

The heat shock proteins (Hsp) are implicated in the survival and cellular thermal tolerance in response to thermal challenge [4,41] and thus were fully characterised *in silico* for these two species using structural and phylogenetic analyses.

### Structure of the HSP70 isoforms

**Fig 2:**
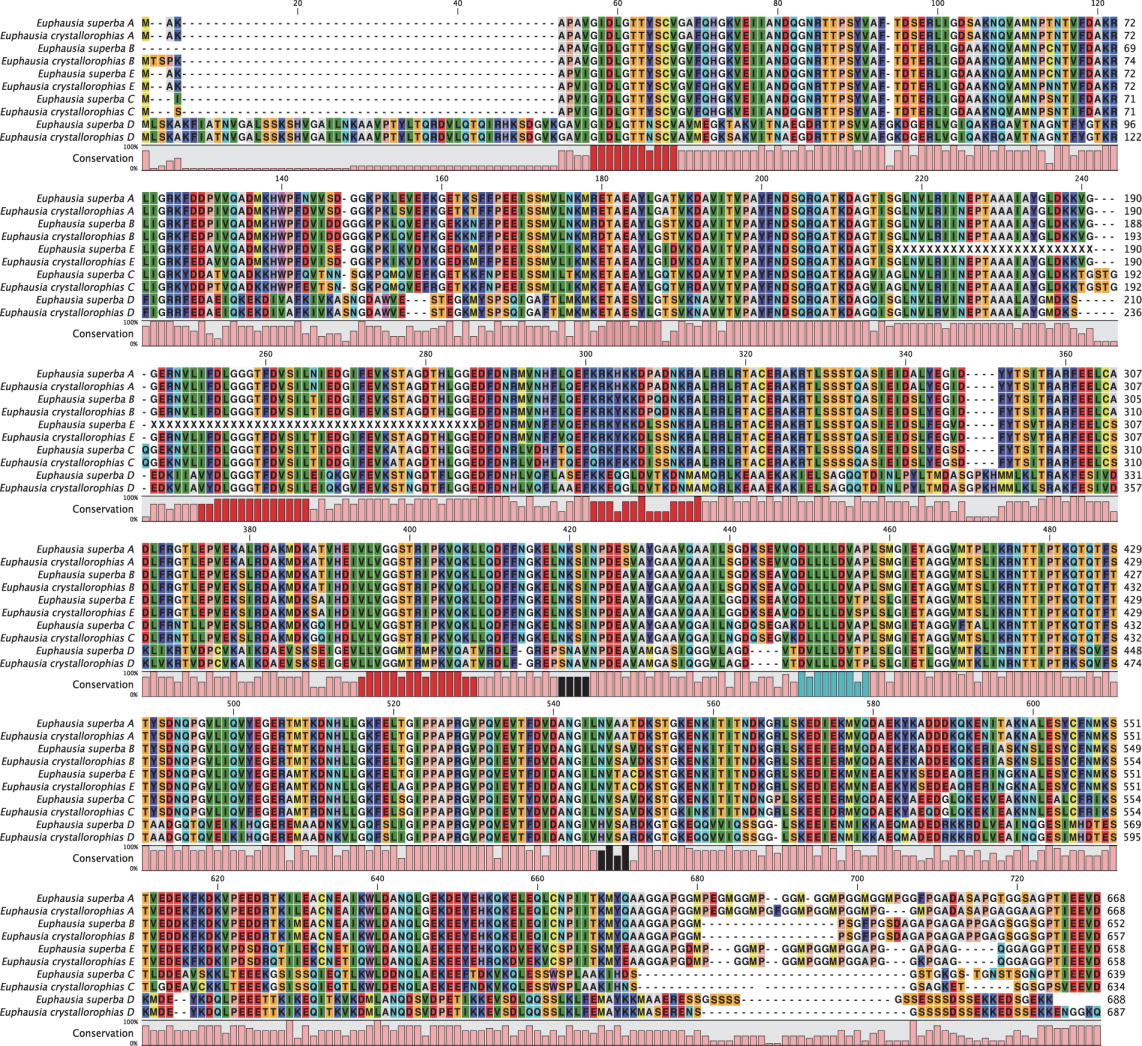
Alignment of five Hsp70 isoforms from *E*. *superba* and *E*. *crystallorophias*. In red: Hsp70 diagnostic motifs; in black: possible glycosylation sites; in blue: hydrophobic linker between Nucleotide Binding Domain and Substrate Binding Domain.

The transcriptome assembly of *E*. *crystallorophias* had a greater sequencing depth and so was more extensive, containing almost full-length sequences for 5 isoforms of *Hsp70* (A to E) (Fig. 2) and *Grp78* (78kDa glucose related protein) (S3 Fig.), a closely related family member, which enabled a subsequent re-analysis of the *E*. *superba* transcriptome, with the identification of 4 additional partial *Hsp70* sequences. There was a very high level of sequence conservation between the two species and this confirmed the existence of 4 putative paralogous genes in each species denoted here as A, B, C and D. Isoform E was not identified in the original analysis of the *E*. *superba* transcriptome, but was partially characterised using RT-PCR approach with heterologous primers designed from *E*. *crystallorophias*. The sequences obtained from the assemblies were confirmed and completed using PCR of cDNA to produce a complete protein sequence for all paralogs, with the exception of isoform E in *E*. *superba* (Fig. 2). All sequences contain the three motifs and the two principal regions of the molecule (the Nucleotide Binding Domain (NBD) and the Substrate Binding Domain (SBD) joined via a linker) [42], which are diagnostic of the Hsp70 family (Fig. 2). These are particularly well conserved in the A, B, C and E forms and more variable in the D form.

The different isoforms were very similar to each other at the amino acid level with identities ranging from 78% to more than 99% between the isoforms (S2 Fig.), with the exception of isoform D. The latter was clearly very different with a relatively low amino acid sequence identity of only 49–51%, when compared with the other Hsp70s. The calculated protein mass of this isoform in both species was also higher than the other Hsp70’s, at 74.5 kDa and therefore should probably be re-named as Hsp74. The best matches using Blastx sequence similarity searching were found with insect sequences designated as a mitochondrial form; Hsp70 cognate 5 and thus isoform D is similarly putatively designated as a mitochondrial HSP70 family member.

The first four isoforms (A, B, C, E) all have a common terminal motif (I/VEEVD) indicating their cytoplasmic localisation. Also the A, E and to a lesser extent B contain the tetrapeptide repeat motif (GGMP), which is present once in the B form in the two species, 4 or 6 times for the A and 3 times for E form (Fig. 2). This repeat sequence has been implicated in the interaction with a co-chaperone protein [43] and is characteristic of the constitutive forms [44]. This motif is absent from the C isoform. This sequence aligns closely with the inducible form of other crustacean HSPs, such as HSP70_1 and HSP70_2 of *Rimicaris exoculata* [45], HSP70_1 of *Palaemonetes varians* [46] and that of *Fenneropenaeus chinensis* [47]. These alignments demonstrate the absence of the tetrapeptide GGMP at the extreme C terminal of all the isoforms and the addition of an extra well-conserved 2–4 amino acids at this location (aa 191–194 in cytosolic krill isoforms). Since the inducible nature of these isoforms has been experimentally demonstrated in these different decapods, it was logical to hypothesize via sequence homology, the potential inducibility of the C isoform in krill.

From current data, it was notable that there is usually one form of inducible Hsp70 for two or three constitutive and this is the case at the cytoplasmic level (isoforms terminating with the motif EVD) in krill. In *Rimicaris exoculata* [45,46], where four *hsp70* genes have been characterised, two were shown to be functionally inducible. In the insects, members of the genus *Drosophila* have 2 to 5 genes coding for inducible Hsp70s [48]. The duplication of genes and their loss in certain organisms could represent an adaptive response and support the hypothesis that Hsp70 is a particular target of natural selection [49]. The extreme conditions in the Southern Ocean have almost certainly exerted a selection pressure on this frequently duplicating gene family, resulting in either the maintenance of additional isoforms via sub-functionalisation or alternatively, their deletion (Prince & Pickett 2002). This is substantiated by the finding that the motifs which have been potentially implicated in the interaction with ATP/GTP, the bi-partite nuclear targeting sequence (KRKHKKDPADNKR) and glycosylation sites (NKSI and NVSA) [50] are much more variable between the different forms, indicating potential differences in function. In the Antarctic context, it is reasonable to assume that the presence of “extra” forms can increase the diversity of the Hsp70 response to temporary stresses, whilst ensuring the maintenance of high levels of expression of Hsc70, which are required for the increased challenge of protein folding in such a cold environment [51].

In spite of differences in isolation procedures for Hsp70 in different species, some authors reported similar Hsp70 isoforms, as have been identified in this study. The most notable are those of the crab *Portunus trituberticulatus* [52] and the hydrothermal vent shrimp *Rimicaris exoculata* [45,46]. However, all the isoforms have not been found in all the species, but this may be due to a lack of sequence coverage for each species. To further underline the disparity in sampling, an ortholog of isoform E has only been identified in the Portunidae (ACZ02405.1) to date.

### Molecular phylogeny of Hsp70s

**Fig 3:**
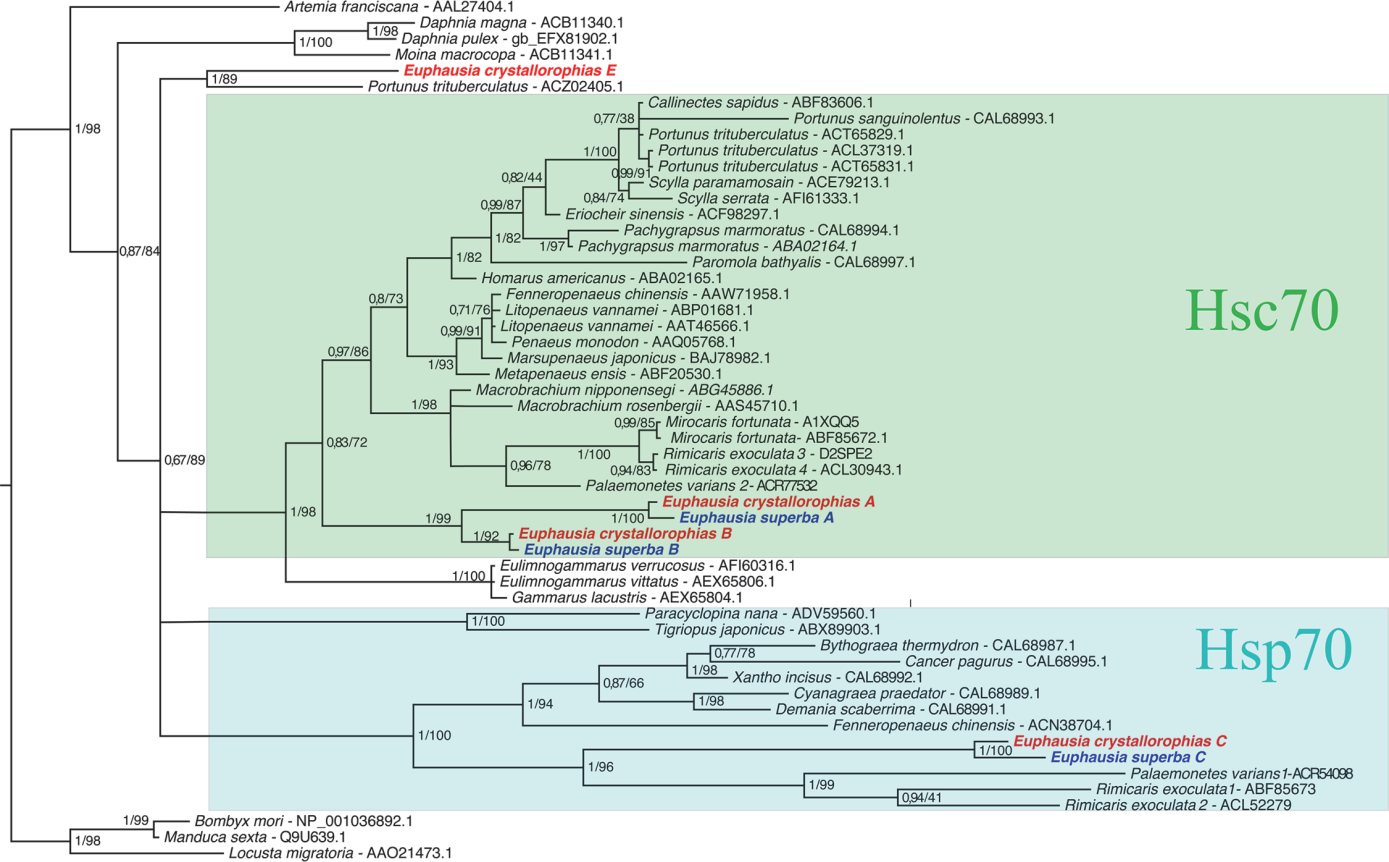
Phylogeny of the HSP70 family in Eucrustacea based on Maximum Likelihood and Bayesian analyses of amino acid data set. Numbers above branches are posterior probabilities and bootstrap values (based on 100 replicated).

In order to determine the phylogenetic relationships between the different Hsp/Hsc70 isoforms in krill, 54 sequences from the Pancrustacea were aligned with the isoforms and a phylogenetic tree produced via both Maximum Likelihood and Bayesian inference methods (Fig. 3). The trees generated by both of these methods were congruent and showed that the position of the *Euphausia* cytoplasmic Hsp70s agreed with previous analyses, as the sister group to the Decapods [53]. It also enabled a distinction between two main groups within the Malacostracea: one cluster contained sequences, which had been structurally determined as Hsc70s, whilst the second group clustered with the Hsp70s [45–47,54,55]. In support of the previous structural analyses using signature motifs, both the A and B isoforms from krill clustered with other Hsc70 genes. A and B isoforms also exist in *M*. *fortunata* and *R*. *Exoculata*, the tree topology indicates that the shrimp isoforms may be allelic variants but not paralogs. However, in the two species of krill, the diversification of the A and B forms clearly arose via a duplication event, which occurred before their speciation.

The E isoform clusters in a position showing that is most probably a sister group to the A and B forms (at a basal position of the two paralogous lines, with the weakest likelihood value of Lli = -9467.6, pREEL = 0.989, from the 5 topologies tested). Thus the evolutionary position of the E isoform (along with the possession of the GGMP motif characteristic of the HSC70 family) indicates that it is potentially close to the ancestral form at the base of the group comprising the Hsc70s within the Crustacea.

The C isoforms in krill group in a second cluster: the inducible Hsp70s. The long branches of this group signify a rapid evolution of these isoforms, especially in comparison with the Hsc70s. However, sequences from the C lineage were splitted into two clades in some species but not in others. It is thus possible to hypothesise that two paralogous lines of isoform C co-occur in the Hsps70s, with the loss of some of these genes according to the taxon. The absence of one of the two Hsp70C paralogs in several crustaceans may either due to a significant difference in expression between the two forms, with one mainly represented (and identified) in transcriptomes to date, or a loss of one of the genes in some lineages. The former is more probable for several reasons: Firstly, the peneid shrimps and krill are the basal groups in each gene cluster. Traditional phylogeny identifies these taxa as being closely related, with the Euphausiacea being the most basal one. Hence if this taxon only contained one of the two paralogs, it is difficult to explain why the second form is only in peneids. Secondly, the branch lengths indicate that within the Caridae and Euphausiacea, these genes have evolved more rapidly with a lighter selection pressure on the duplicated isoforms. Finally, studies in *Drosophila* species, which includes both whole genome and transcriptome data, have established that this genus had ancestrally two *hsp70 C* genes [48].

### Rate of evolution and selection strength within each set of Hsp70 paralogs

The analysis of divergence between the two species, *E*. *superba* and *E*. *crystallorophias* within each set of Hsp70 paralogs clearly showed that they did not evolve at the same rate. Since the time elapsed after the krill species speciation, the lineages of B, C and *grp78* have accumulated more synonymous mutations than those of A and D (cf. dS: Table 1). From this latter table, lineage D evolved at a slower rate when compared to the cytoplasmic ones but lineage C accumulated more non-synonymous mutations than the other lines. This strong heterogeneity of evolutionary rates between paralogs indicated that the Hsp70 genes underwent adaptive evolution since the speciation of the two krill species. In this case, the accumulation of numerous amino acid changes in lineages C and D may be the result of slight functional readjustments of the protein products to adapt to different environments that each krill species inhabit. This contrasts with the other paralogous lineages for which most differences are synonymous. Paralog difference in the synonymous rate (up to 4 times) is associated with the lack of molecular clock in the evolution of this specific multigene family. However, in spite of these differences, due to the intrinsic mutation rate of each paralog, it seems that the gene *hsp70C* has recently evolved under selective relaxation in, at least, one of the two krill species (values of dN/dS 10 times superior to those found in other genes).

**Table 1:** Number of synonymous and non-synonymous mutations between the two krill species.

Gene	N.dN	S. dS
*Hsp70A*	4.0	26.5
*Hsp70B*	2.0	48.0
*Hsp70C*	20.5	38.5
*Hsp70D*	5.0	5.0
*Grp78*	5.0	42.0

N.dN and S.dS estimates obtained between *E*. *superba* and *E*. *crystallorophias* for the 5 paralogous *Hsp70* genes using the Nei-Gojobori method [79] to make evolutionary-rate comparisons under the assumption of a global molecular clock.

A search for positive selection was thus performed using the package CodeML implemented in PaML software (Phylogenetic Analysis by Maximum Likelihood: Yang 2007). Testing the ‘molecular clock’ hypothesis over Hsp70 paralogs was first undertaken by comparing the neutral model M_0_ (identical d_N_/d_S_ between branches) and the models under global (M_0_clock_) and local (M_0_local clock_) clocks. The LRTs (Likelihood Ratio Test) obtained (global: 8.9, local: 5.8) could not discriminate between the two hypotheses (non-significant Chi^2^: Table 2), even if the model without clock fits the sequence data better. This indicates that paralogs are not evolving at the same speed on terminal branches leading to the krill species (no global molecular clock). As the number of mutations accumulated between the two sister species was not shared equally (i.e. absence of clock), it is worth noting that at least one of the two species conserves the ancestral type (*HspA*: *E*.*superba*, *HspB*, *C*, *D* and *Grp78*: *E*. *crystallorophias*), with the other krill species clearly diverging from this original form.

**Table 2:** Parameter estimates of the selection models implemented in the CodeML package of PaML vs 3.44 [31], likelihoods (LnLi) and associated likelihood ratio tests (LRT).

**Model**	**LnLi**	**p**	**Model estimates**	**LRT (df)**	**Sites under positive selection (BEB>0.90)**
**Branch Models**					
M_0_	-9467.6^$^	22	ω = 0.021		
M_0__local clock	-9470.5	14	ω = 0.021	5.8 ^NS^ (7)	
M_0__clock	-9472.0	12	ω = 0.021	8.9^NS^ (9)	
M_1_	-9411.8	41	0.0001<<999	111.6*** (19)	EusHsp70A^&^EucHsp70D^&^EucGrp78^&^
**Site Models**					
M_1A_ ‘nearly neutral’	-9414.0^#^	23	ω_0_ = 0.015 (93.9%)		
			ω _1_ = 1 (6.1%)		
M_3_ ‘selection’	-9414.0	25	ω_0_ = 0.015 (93.9%)	0^NS^ (2)	
			ω _1_ = 1 (6.1%)		
			ω _2_ = 1 (0%)		
**Branch-site Models**					
M_2A– EusHsp70A_	-9414.0	25	ω_0_ = 0.015 (93.9%)	0^NS^ (2)	
			ω _1_ = 1 (6.1%)		
			ω _2a_ = 1 (0%)		
			ω _2b_ = 1 (0%)		
M_2A– EusHsp70C_	-9398.3	25	ω_0_ = 0.012 (92.0%)	31.3* (2)	^129^KR,^480^EI,^497^ED,^509^KQ
			ω _1_ = 1 (5.6%)		
			ω _2a_ = 999 (2.2%)		
			ω _2b_ = 999 (0.2%)		
M_2A– EucHsp70C_	-9394.6	25	ω_0_ = 0.011 (92.9%)	38.6** (2)	^392^MF,^394^SA,^525^SA,^569^KT
			ω _1_ = 1 (5.7%)		
			ω _2a_ = 999 (1.3%)		
			ω _2b_ = 999 (0.1%)		
M_2A —EucHsp70D_	-9413.9	25	ω _0_ = 0.013 (0.2%)	0.2^NS^ (2)	The two Hsp70D are highly divergent & saturated
			ω _1_ = 1 (6.1%)		
			ω _2a_ = 1 (87.5%)		
			ω _2b_ = 1 (6.2%)		
M_2A– EucGrp78_	-9404.2	25	ω = 0.015 (93.3%)	19.6* (2)	
			ω _1_ = 1 (6.1%)		
			ω _2a_ = 1 (0.5%)		
			ω _2b_ = 1 (0.1%)		

p: number of parameters estimated by the model, model estimates: ω = dN/dS with 4 classes: ω_0_ = ω<1, ω_1_ = ω = 1,ω_2a_ = ω>1against ω_0_ andω_2b_ = ω>1 against ω_0._ (df): degree of liberty

NS: not significant

*: significant at 0.05

**: highly significant at 0.01

***: very highly significant at 0.001

$: null hypothesis used in the LRT for branch models

#: null hypothesis used in the LRT for site- and branch-site models and branches under positive selection.

**Fig 4:**
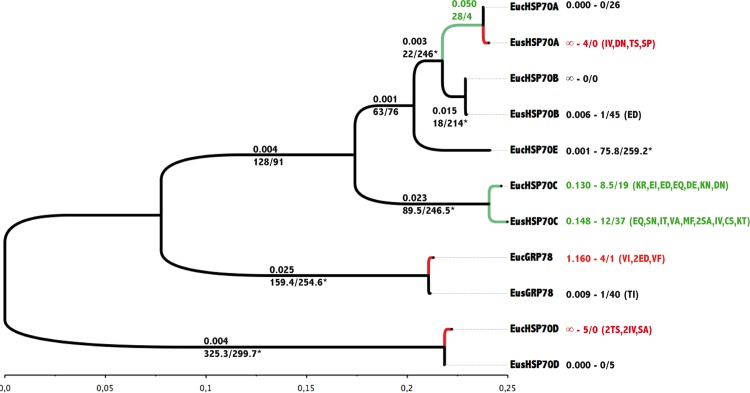
Non-synonymous (dN) tree of the 11 Hsp70 genes found in *E*. *superba* and *E*. *crystallorophias* obtained using the free-ratio (M1) branch model of CodeML according to the Goldman & Yang (1994) model of codon substitution. Above internal branches: dN/dS (ω) ratio, below internal branches: number of non-synonymous and synonymous mutations estimated from the ancestral sequence reconstruction. * indicates saturation at the synonymous sites. The green colour represents branches under putative selective relaxation and the red one, branches (mostly terminal) under positive selection. Next to external (terminal) branches also corresponds to the ω ratio and the number of non-synonymous and synonymous mutations obtained from the ancestral sequence reconstruction using the M_2A_ branch-site model of selection. If any, a list of amino-acid replacements that corresponds to positively selected sites with a BEB probability greater than 90% is provided together with these values.

To evaluate, in finer detail, the role of diversifying selection in the evolution of the Hsp70 family in krill, an analysis was then performed on the evolution of non-synonymous to synonymous ratio (dN/dS) both between the branches leading to each paralog and codons of the *hsp70* sequences by fitting sequence datasets to a model of codon substitutions developed by Goldman & Yang (1994) under different hypotheses of evolution, either neutral or selective. Comparisons between models are shown in Table 2. The selection Branch model M_1_ (dN/dS free to vary between branches) was clearly better than the nearly-neutral model, M_0_ (a single dN/dS for all the branches: lnL = -9411,8: Table 2) with several branches displaying d_N_/d_S_ values greater than 1 (Fig. 4). Although the majority of internal branches were saturated (rate of synonymous replacements > 50% of the sites), this tree showed that most of the non-synonymous diversification had an early influence on the 3D-structure and function of these proteins at the time the paralogs first separated. It is however interesting to note that terminal branches, which correspond to Hsc70C have undergone selective relaxation in both polar species (Fig. 4). The reconstruction of ancestral characters via a Bayesian approach was only reliable in the most recent nodes and especially at the level of the ancestral nodes giving rise to the two species. This approach showed that *E*. *superba* accumulated many more silent mutations than *E*. *crystallorophias*, but unexpectedly nearly all of the mutations found within this latter species are amino acid replacements (all terminal branches except *HspA*: Fig. 4), leading to the conclusion that this evolution should be of an adaptive nature. The reconstruction of ancestral characters when constrained by a global molecular clock weakly affected the distribution of non-synonymous mutations between the two species (only one replacement exchanged between the 2 species in paralog C (Cys->Ser in Es vs Ser-> Cys in Ec) while the number of synonymous mutations re-equilibriated between the terminal branches.

The analysis of codons under positive selection by comparing the positive selection Site model M_3_ to the almost neutral model M_1A_ across paralogs rejected the hypothesis of positive selection on a specific codon (Table 2). However, because selective relaxation was suspected in the specific case of the C lineage in the 2 krill species, a more detailed search of codons under positive selection was undertaken to specifically compare the evolution of codons in either *E*. *superba* or *E*. *crystallorophias* on terminal branches exceeding a value of ω>0.25 by considering the codon evolution on this branch (forward branch) against the whole tree (‘background’ branches). The analysis was carried out by comparing the Branch-Site selection model M_2A_ to the nearly-neutral model M_1A_ (Table 2) and showed that only the paralogs C (*E*. *superba* and *E*. *crystallorophias*) and *Grp78* (*E*. *crystallorophias*) fit the selection model (M_2A_). In parallel, ancestral sequence reconstruction suggested that several codons were under positive selection (Bayesian probabilities >90%). These codons were uniquely found in paralogs C and more specifically favoured replacements, which lead to a loss of charge at the protein level (eg. EI or KN) or towards an increase in the degree of hydrophobicity (eg. SA or MF). Such changes have previously been described as a mechanism for molecular adaptation to cold and act by diminishing the rigidity of the proteins [56,57]. This trend was particularly marked in *E*. *crystallorophias*.

The duplication of genes has played an important role in the evolution of species and the development of biological complexity in the Metazoa [58,59]. This process provides the principal evolutionary force behind the acquisition of new functions (process of neo-functionalization [60,61] or a specialisation of tissue- or developmental-specific isoforms and/or division of cellular tasks (process of sub-functionalization [62]). These processes can be identified in the Hsp70 multigene family, which has an evolutionary history rich in duplication events. The study of these proteins in krill revealed an astonishing diversity of isoforms. These were the result of, at least, five duplication events in an organism designated as being at the base of the Malacostraca crustacean group, which has evolved in the polar environment with specific adaptations to cold [12,13]. The increase in the number of copies of a gene by successive duplications, is often adaptive, notably in terms of adaptation to low and high temperatures when such a duplication is relatively recent [63]. However, the extreme stability of the southern environment for millions of years [64] could have led to the loss of inducibility of these proteins as has already been described in other Antarctic coastal species [4,65,66] or the loss of the gene itself. In krill the number of substitutions accumulated between the different paralogous lineages shows that this diversification was early in the evolution of the Arthropods, with sub-functionalization evident in the different isoforms. This is characterised by the strong accumulation of non-synonymous mutations in the D lineage and GRP78, which together show a diversification of functions and compartmentalisation to particular cellular environments, along with specific diagnostic amino acid signatures [43,44]. The divergent evolution of the five paralogous lineages with each evolving under strong purifying selection reinforces the idea of sub-functionalisation of these genes [63] with the fixation of “adaptive” mutations over time, and the lack of detectable positive selection because of a saturation, evident among synonymous sites.

However, the Hsp70 lineage C, designated as an inducible cytosolic form, exhibits a specific evolutionary history in the two species of Antarctic krill. This paralog indeed shows a selective relaxation after the emergence of the 2 krill species, and potential for positive selection on certain types of amino acid replacements. Results therefore lead to the development of two hypotheses as potential explanations for the specific evolutionary history of lineage C in krill. The first explanation could be the recent degenerate evolution of this isoform towards a pseudogene (i.e. fixation of deleterious mutations), simply because inducibility may be no longer necessary in a stable environment where the temperature rarely varies above -1.5°C. However this is unlikely since the protein is still functional and because the relaxation of selection seems to pre-date the speciation event leading to the formation of the two species several millions of years ago. Even if the gene was perhaps maintained by gene conversion via beneficial mutations (Fawcett and Innan, 2011), the time elapsed since the separation of the paralogs A, B and C appears too deep in history to support this. One could nevertheless imagine that a new duplication could have arisen recently in lineage C just before the species separation in order to enhance phenotypic plasticity [67,68]. Such a scenario would however only be detectable via an analysis of the *Hsp70* genetic diversity within and between the two species. The second explanation is that the inducible isoform may have evolved towards a constitutive form leading to a higher efficiency of the chaperon at lower temperatures. This could enable this isoform to respond more efficiently to cold stress when the animal is on the point of freezing. This hypothesis is supported by the fact that in the two species of krill, notably *E*. *crystallorophias*, the majority of amino acid replacements under positive selection turns charged amino acids into polar ones. Species living in cold environments are likely to display these types of replacements, which in general, confer increased flexibility on the protein and therefore an increased catalytic efficiency at low temperatures [56,57]. This trend appears particularly marked in the ice krill (*E*. *crystallorophias*) and could, therefore, be related to its lifestyle in the colder coastal waters of the Antarctic and its over wintering under the sea ice [17].

### Kinetics of Hsp70 expression

**Fig 5:**
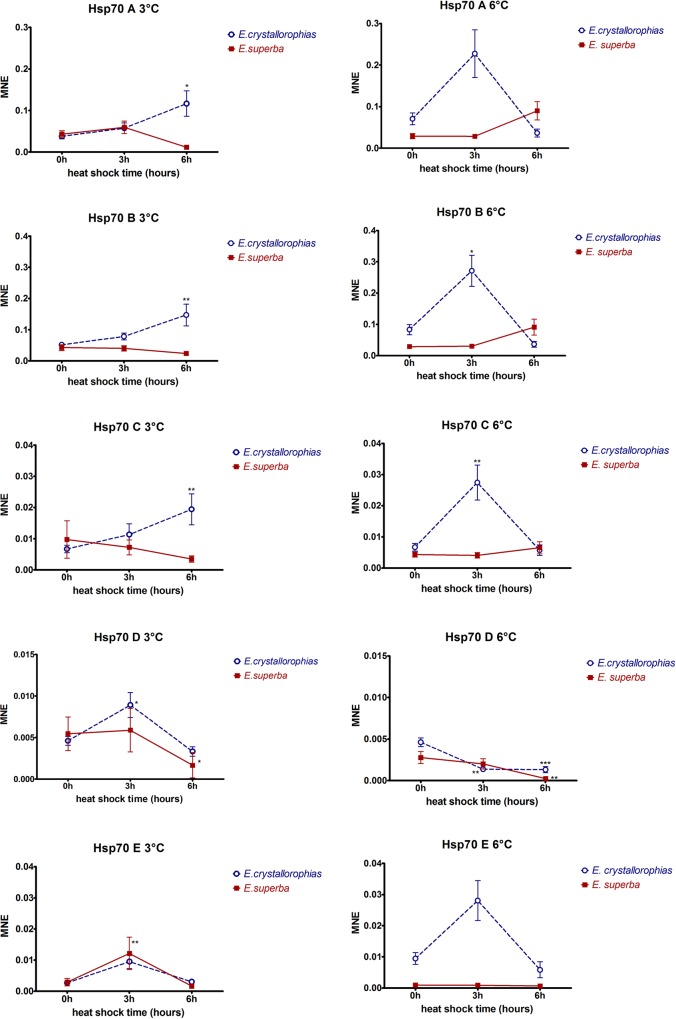
Expression levels of *hsp70* genes obtained by qPCR in two krill species: *E*. *superba* (red solid line) and *E*. *crystallorophias* (blue dotted line) during a heat shock of 3°C and 6°C whereas 0h group is control at 0°C. Heat shocks were carried out during 3h and 6h, and hsp70 expressions measured at those time on n = 6 to 10 individuals per group. Hsp70 expressions for both species were normalized by the *18S* gene expression. To compare values, a Kruskal-Wallis test followed by a Dunn’s test were used to compare control groups (0h) and shocked groups (3h and 6h). Significant differences between mean normalized expressions are indicated by asterisks (*).

The kinetics of expression of the different isoforms was analysed in both species in response to moderate thermal shocks of 3°C and 6°C. These temperatures were chosen in order to be compatible with possible scenarios of global warming (Fig. 5). The level of expression in control animals for each of the isoforms was different and suggested that the basal level of expression strongly depends on each isoform, providing further evidence for the sub-functionalization hypothesis. Both the molecular and phylogenetic analyses have enabled predictions to be made with regard to the status of the different isoforms, such that Hsp70A, B and E are potentially constitutive, Hsp70C is inducible and finally Hsp70D is mitochondrial. If A and B represent the constitutive forms, their basal expression is expected to be superior to the inducible form (C), as it is the case (Fig. 5). The forms D and E are less highly expressed. These observations on basal expression levels are validated by comparison with the FPKM values from the transcriptome analysis of *E*. *crystallorophias* (Table 3).

**Table 3:** *E*. *crystallorophias Hsp70* isoform expression values and associated Blast matches.

	**Comp ID**	**Size (aa)**	**Size (pb)**	**FPKM**	**BLAST matches (E-value)**
***EucHsp70A***		**668**	**2007**	**395.1**	Hsc70 *Mirocaris fortunata*
	comp586_c0_seq1	179	538	254.2	(0.00) ABF85672
	comp1674_c0_seq2	178	535	52.3	
	comp1806_c0_seq1	177	532	88.6	
***EucHsp70B***		**657**	**1971**	**404.6**	Hsc70 *Litopenaeus vannamei*
	comp208_c3_seq1	657	2281	404.6	(0.00) AAT46566
***EucHsp70C***		**634**	**1902**	**33.2**	Hsp70 *Metapenaeus japonicus*
	comp2276_c0_seq1	634	2061	33.2	(0.00) BAJ78982
***EucHsp70D***		**687**	**2061**	**16.2**	Hsp70 *Tribolium castaneum*
	comp3534_c0_seq1	687	2791	16.2	(0.00) XM_970293
***EucHsp70E***		**658**	**1974**	**22.5**	Hsp70 *Portunus trituberculatus*
	comp17582_c0_seq1	152	1012	5.6	(0.00) ACZ02405
	comp2807_c0_seq2	509	1653	16.9	

comp ID: sequences assembled with Trinity. Size (aa): deduced coding sequences. Size (pb): comp or contig sizes in pair bases. FPKM = Fragments Per Kilobase of exon per Million fragments mapped. Values of the isoform TPM or FPKM are in bold.

The rapid transition from 0°C to 3°C did not invoke an up-regulation of *Hsp70* in *E*. *superba*, in fact there was a general trend for a reduction in gene expression after 6 hours compared with at time zero. The response of *E*. *crystallorophias* was clearly very different, showing an increase in expression after 6 hours for the A, B and C isoforms. The heat shock at 6°C did not provoke a significant response after 3 hours in *E*. *superba*, but with a slight increase in expression by 6 hours for transcripts A, B and C (Fig. 5). The responses of isoform A, B, C and E in *E*. *crystallorophias* were very different, with an increase in expression at 3 hours, which diminished by 6 hours. The expression pattern whereby there is a peak at 3 hours followed by a return to a value below that of the original could be due to a feedback loop of the Hsp70 to their original expression, and provides evidence of the limited Hsp70 response in these species.

It is evident from the expression kinetics that responses vary not only between the isoforms, but also between species. When a statistically significant response was detected, the increase in absolute levels was very low. In most of the studies conducted on Antarctic species, the animals (with most examples from benthic invertebrates) were subjected to an acute thermal shock greater than that described here (+10–+15°C). Whilst these temperature increases are not representative of warming which may occur in the environment, it took such dramatic challenges to induce a heat shock response in the first place and not all of the species were capable of mounting this response [69]. This study reported here is the first to document the response to thermal challenge in pelagic invertebrates. The level of up-regulation of *Hsp70* expression in these two krill species is not large, varying between zero and up to a maximum increase of three-fold. These results indicate that the ability to up-regulate these proteins has not been lost, but is minimal and this is not exceptional for species living in very cold and stable environments when considering environmentally relevant challenges [70,71]. It is certainly much lower than that observed in animals from temperate regions where the increase in expression can be many times superior for certain isoform of Hsp70 [55].

In both experiments at 3°C and 6°C, there was no difference either in profile or in amplitude of the response between the four cytoplasmic isoforms (A, B, C, E), which goes against the paradigm that Hsc70s and Hsp70s are constitutive versus inducible respectively. What is clear from these expression experiments is that, although the functions and levels of activity must be different, the notions of inducibility and constitutivity do not appear to be operating here. The A and B isoforms have a structure typical of Hsc70s and cluster with other Hsc70s in phylogenetic trees. Moreover, they have a high basal level of expression, and show a significant over expression particularly in *E*. *crystallorophias* and to a lesser extent in *E*. *superba*. The C forms, which show all the sequence characteristics of an inducible Hsp70 in terms of their structure and phylogenetic position, had a weak basal expression level and behaved like the A and B isoforms, with similar fold increases in expression. Thus, in spite of its inducible signature motifs, the expression data of *Hsp70C* are not compatible with such a functional designation, especially not in *E*. *superba*, implying that inducibility may have been lost and could potentially explain why a relaxation in selection pressure has been detected with this paralogous sequence. The question of whether an *hsp70* gene is inducible or constitutive is almost certainly related to the stress invoked, as despite sequence motifs designating a gene as an *hsc70*, induction can occur under certain stresses, as has been shown in another Antarctic species, the limpet *Nacella concinna* in response to long-term acclimation at +2°C or air exposure (Clark and peck, 2009b). It is also obvious that the basal level of Hsp70C is lower than that of A and B. This is in line with other Antarctic species, where a higher level of constitutive Hsp70 expression is required to overcome the problems of protein folding in the cold (Clark and Peck, 2009a) and this is of great interest, particularly with regard to the evolutionary history of this gene family.

The D isoforms are the mitochondrial forms of Hsp70 or mtHsp70. Although they have been little studied in the invertebrates, with the exception of *Caenorhabditis elegans*, they have been shown to have multiple effects in the mitochondria [72–74]. Their mitochondria-specific activity, may explain their modest expression levels with regard to isoforms A and B, and their very different expression pattern, even though they are no less fundamental to the survival of the cell. Recent studies in yeast have confirmed the importance of mtHsp70 in the housekeeping of molecular aggregates prejudicial to the health of the mitochondria in physiological normal conditions. This protective effect would be diminished during a thermal challenge because of the increase in aggregated polypeptides generated by the heat shock and/or a sensitivity of the mtHsp70 itself [75]. Our study shows that, similar to the effects of thermal challenge observed on mtHsp70 in yeast, *Hsp70D* does not see an increase in expression levels in response to a thermal shock. This could lead to a lack of effectiveness in being able to efficiently manage the consequences of this stress on the mitochondrial proteins.

Structurally the E isoforms have been designated as constitutive and cytoplasmic. The representation of these isoforms within the RNA populations of the two species is weak, as seen by the low FPKM in the transcriptome of *E*. *crystallorophias* (Illumina), the absence in the transcriptome of *E*. *superba* (454) and the difficulty of amplifying them via RT-PCR in the *E*. *superba*. These low quantities could explain the poor representation of this specific isoforms within the available databases of Hsp70 sequences in the crustaceans. This isoform is clearly different from the other cytoplasmic forms and does not fulfil the same functions. This outlines the importance of identifying a full set of Hsp70 isoforms for each species, rather than examining each in isolation, thus gaining a better comprehension of the global Hsp70 response in a species [76].

## General Discussion and Conclusions

The two species of Antarctic krill have very separate adult geographic distributions, which may be related to the abiotic profiles of each particular habitat. This potentially impacts their physiological functioning and resilience in the face of climate change. Temperature is a strong candidate for one of the segregating factors influencing the separation of the two species, as one is epipelagic, coastal (*E*. *crystallorophias*), the other is deep sea pelagic (*E*. *superba*). The determination of the temperature limits (CT_max_) of the two species showed a weak, but significant difference to a rapid increase in temperature, with *E*. *crystallorophias* being the most sensitive. These CT_max_ temperatures of circa 15°C are nevertheless more elevated than other polar crustaceans and Antarctic invertebrates, subjected to the same temperature increase and therefore krill appear (using this metric) to be one of the more resilient polar species to warming oceans.

The Hsp70 is a primary biomarker of choice for thermal stress, which is substantiated by the considerable bibliography on this family of chaperone proteins and their response to stress. However, these genes may not be appropriate biomarkers for Antarctic species, as an absence of the classical heat shock response has been noted in several Antarctic fish species and also some invertebrates [4,65,69,77]. The Antarctic krill species studied here do have a heat shock response, in terms of up-regulation of HSP70 family members, although the response is weak compared with the fold changes observed in temperate species. The response of each HSP70 gene is very different for the two species, with a much earlier response to temperature (at 3°C) in *E*. *crystallorophias* and a negligible response in *E*. *superba*. This observation poses the question in this species, of the existence of a cold stress at temperatures lower than or equal to 0°C and to a thermal preference close to 3°C, as many populations localised in the sub-Antarctic region around South Georgia regularly live in waters around 4–5°C [22,78].

The expression data is highly valuable in shedding light on the functionality of the isoforms, with the differing responses of each confounding the nomenclature of inducible and constitutive. The corruption or diversification of the response of the cytosolic constitutive forms to thermal challenge could also be at the origin of the relaxation of purifying selection pressure observed on the “inducible” form C, which may even lead to its eventual change in function and potentially inactivation as a pseudogene.

Overall, the results of these different experimental approaches investigating the thermal limits of two krill species have shown that they have different responses to temperature. Therefore this abiotic factor could play a significant role, not only in their geographical separation, but also their relationship to sea ice. The sensitivity of the epipelagic species *E*. *crystallorophias* to variations in temperature and/or its adaptation to cold appears to be more critical than with its sister species *E*. *superba*. These characteristics correlate with their distributions and life styles. These results indicate that ice krill could be the first one to be impacted and may represent a case of “loser” species as previously defined by Somero [10], when coastal waters will warm in the coming years due to climate change, irrespective of any other biotic factors that could come into play in the future.

## Supporting Information

S1 Fig Differential-swimming behaviours of the two krill species during CT^max^ experiments. Animals present in the water column above the bottom are only considered. *E*. *superba* in red and *E*. *crystallorophias* in blue.
(TIF)

S2 Fig Percentage of identity between krill amino acid and nucleotidic Hsp70 sequences. (TIF)

S3 Fig Alignment of Grp78 isoforms from *E*. *superba* and *E*. *crystallorophias*. In red: Hsp70 diagnostic motifs.
(TIF)
