# Supplementary figures and images for: Diversification, Evolution and Sub-Functionalization of 70kDa Heat-Shock Proteins in Two Sister Species of Antarctic Krill: Differences in Thermal Habitats, Responses and Implications under Climate Change
Source: PLoS One. 2015 Apr 2;10(4):e0121642. doi: 10.1371/journal.pone.0121642 (PMC4383606; doi:10.1371/journal.pone.0121642)

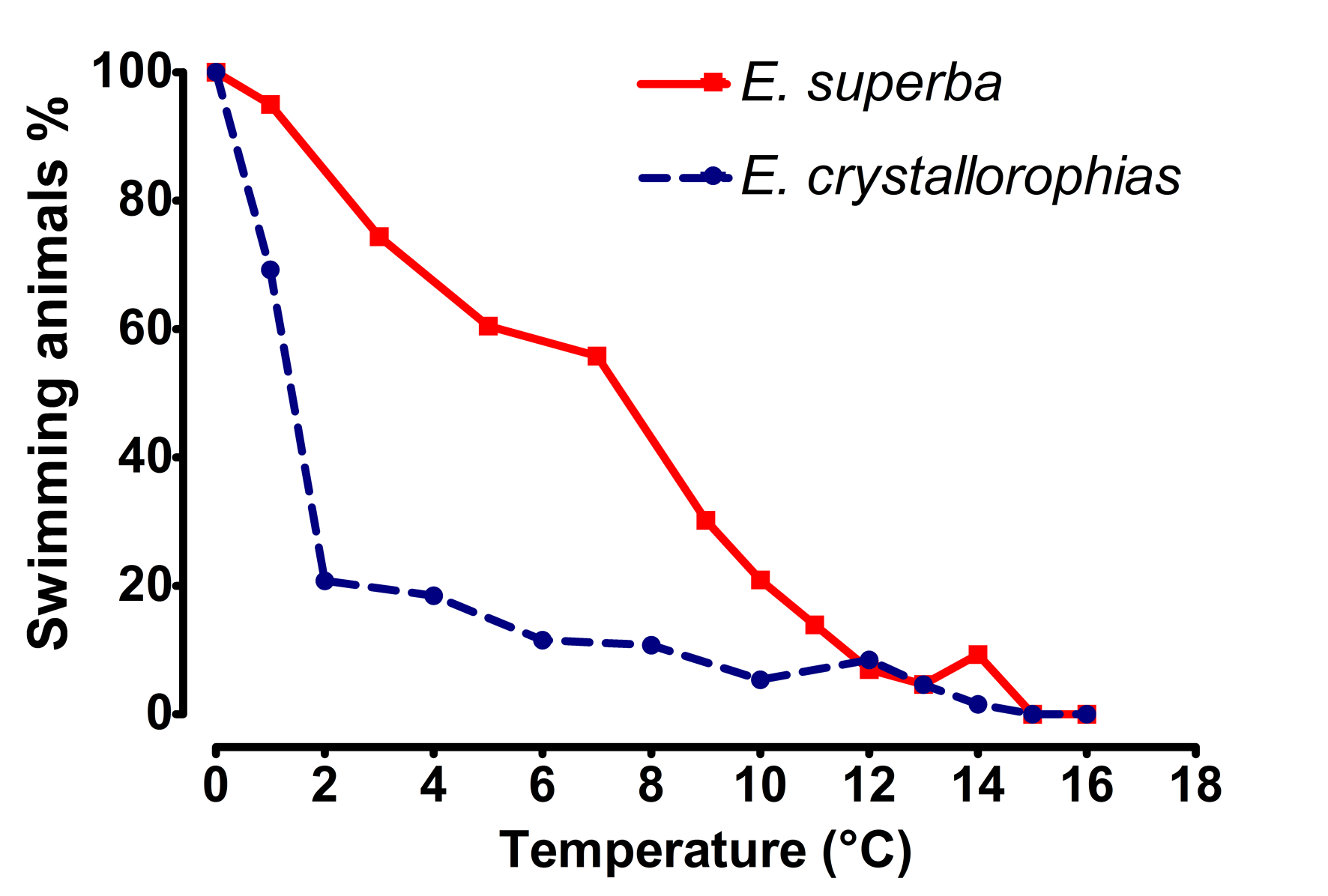

Supplement: S1 Fig — Animals present in the water column above the bottom are only considered. E. superba in red and E. crystallorophias in blue. (TIF) [file pone.0121642.s001.tif]

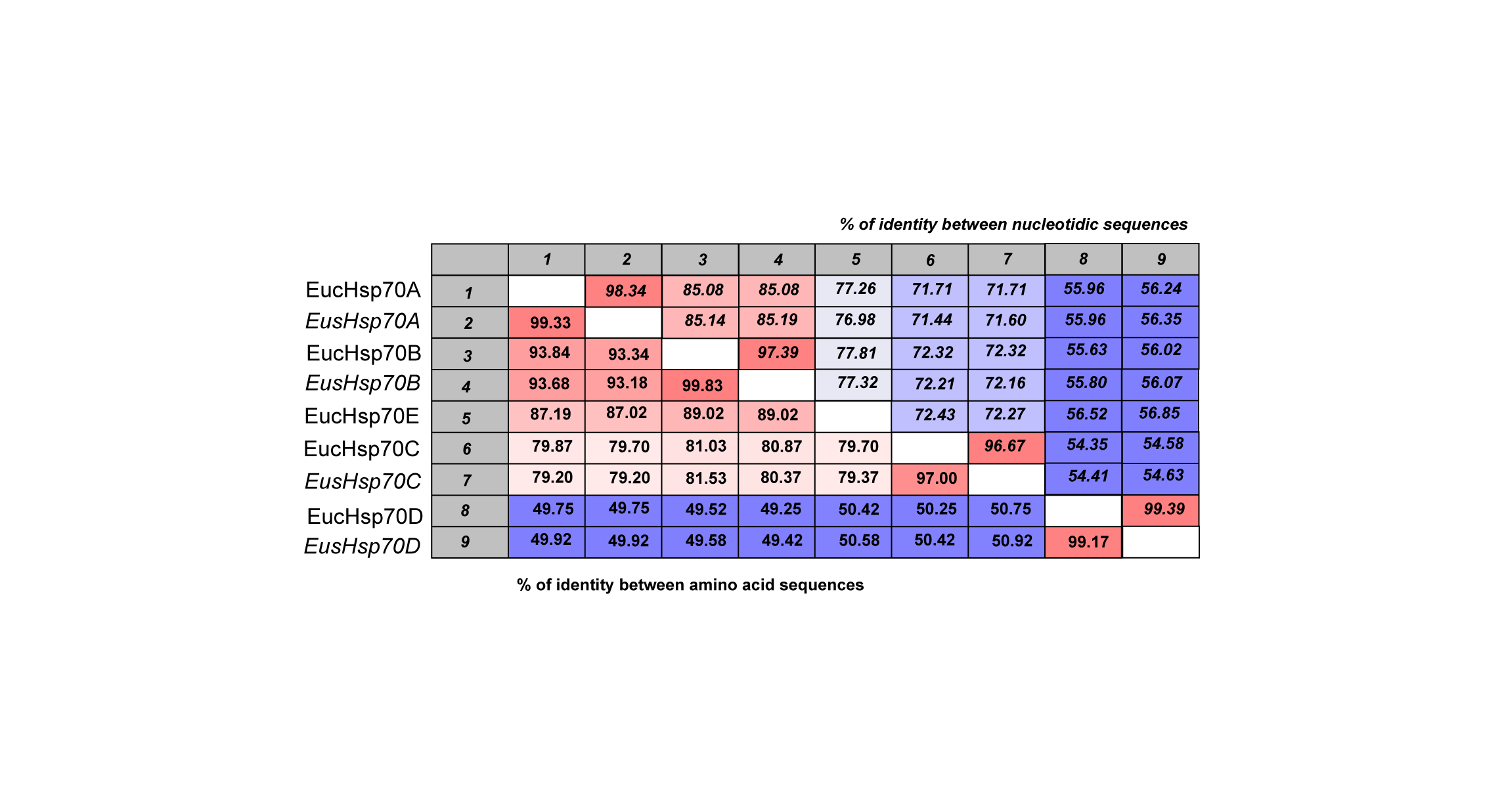

Supplement: S2 Fig — (TIF) [file pone.0121642.s002.tif]

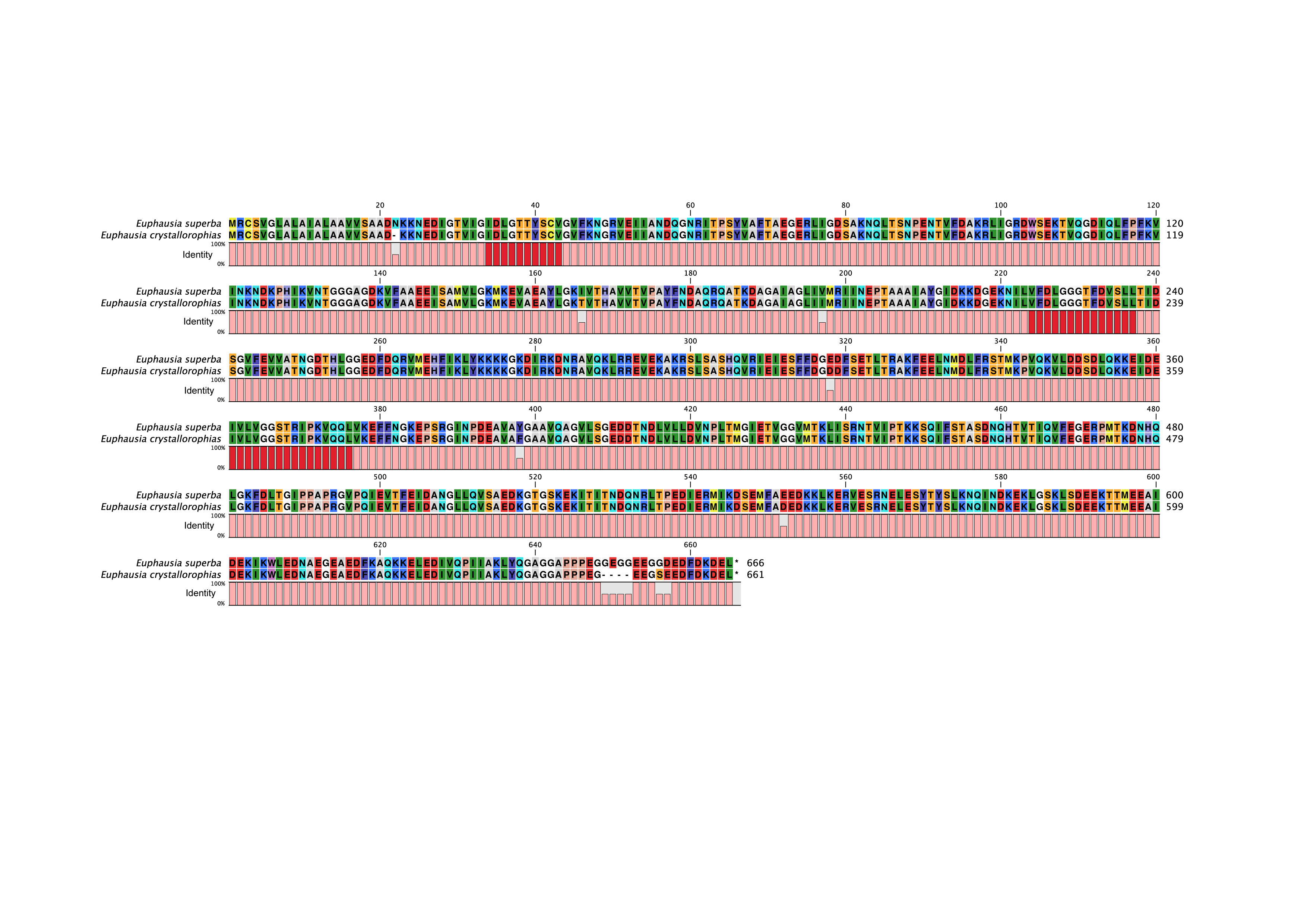

Supplement: S3 Fig — In red: Hsp70 diagnostic motifs. (TIF) [file pone.0121642.s003.tif]
